# Omission and Compromise: The Sacredness of Moral Foundations in Political Groups in Italy

**DOI:** 10.5964/ejop.v16i1.1887

**Published:** 2020-03-03

**Authors:** Silvia Di Battista, Monica Pivetti, Annukka Vainio, Chiara Berti

**Affiliations:** a Department of Psychological, Health, and Territorial Sciences, University “G. d’Annunzio” of Chieti-Pescara, Chieti, Italy; b Department of Forest Sciences, Faculty of Agriculture and Forestry, Helsinki Institute of Sustainability Science (HELSUS), University of Helsinki, Helsinki, Finland; University of Wroclaw, Wroclaw, Poland

**Keywords:** moral psychology, compromise on moral foundations, omission, sacred values, moral foundations, political orientation

## Abstract

Sacred values are moral foundations that may make public and political debates among groups hard to resolve. A taboo trade-off framework offers the opportunity of measuring the inviolability and the “sacralization” of moral foundations. In this study, moral foundations in a taboo trade-off framework were assessed in a convenience sample of Italians (N = 224) using a new measure to assess sacred values, the Omission as a Compromise on Moral Foundations scale (OC-MF). The OC-MF measures the willingness of individuals to omit moral foundations in exchange for money. It was predicted that Italian center and left-wing participants would be less willing to compromise individualizing moral foundations as opposed to binding ones, and that center and right-wing participants would be less willing to compromise on binding moral foundations than left-wing participants. Confirmatory Factor Analyses demonstrated the two-factor structure of the OC-MF: individualizing and binding. As predicted, Repeated Measures Anova showed that political orientation was related with differential adoptions of moral foundations as sacred values, with center and left-wing participants refusing to compromise more on individualizing than on binding moral foundations. Moreover, left-wing participants were more willing to compromise on binding moral foundations than center and right-wing participants. The OC-MF shows the hypothesized differences between Italian political groups and offers a new understanding of moral reasoning. These findings provide opportunities for improving ideological debates concerning sacred values.

## The Moral Compromises of Sacred Values

In many situations, people dislike making a trade-off between different choice alternatives, especially if choice options mirror inviolable sacred values (Tetlock, Kristel, Elson, Green, & Lerner, 2000). Any moral value that people implicitly or explicitly treat as having infinite or transcendental meaning that prohibits comparisons, trade-offs, or other exchanges is referred to as a “sacred value” (Bartels, Bauman, Cushman, Pizarro, & McGraw, 2015; Tetlock et al., 2000).

Graham and Haidt (2012) argued that sacredness is crucial for understanding morality and it is a universal human propensity to invest people, places, times, and ideas with an importance which goes far beyond the utility they possess. In particular, some goods, such as human life, rights, or health, are treated as having a transcendental or sacred meaning that cannot be exchanged for secular values, especially those goods that can be bought or sold (Bartels et al., 2015; Carmichael, Hubert, Reeves, & Schanche, 1994). Sacred values are not always religious in character, because core secular values can be assumed as sacred and inviolable by a community (Graham & Haidt, 2012; Tetlock et al., 2000). Graham and Haidt (2012) explained that sacredness does not require a God, because flags, national holidays and many other markers of collective solidarity are sacred in the same way.

The opportunity for moral compromise of sacred values occurs whenever an individual confronts a situation where a contemplated choice or action puts at risk those moral principles with which an individual and his group strongly identify (Goodstein, 2000). Moral compromise involving what is sacralized are often resisted or refused (Graham & Haidt, 2012). Previous research has shown that trading off sacred values is considered a taboo and people express negative feelings even when contemplating this mere opportunity (Tetlock, 2003). For instance, Hanselmann and Tanner (2008) argued that taboo trade-offs are negatively emotion-laden, because the decision-makers realize that something important is at stake and has to be protected. Authors have also found that when a decision situation is associated with a sacred value (i.e., taboo trade-off), people perceive the task as easier to solve compared with a situation not involving sacred values (i.e., routine trade-off). Performing a functional MRI (fMRI) with volunteers who were faced with a series of decision tasks, Duc, Hanselmann, Boesiger, and Tanner (2013) found that taboo trade-off proposals increased neuronal activations in the left anterior temporal lobe and bilateral amygdalae – areas associated with rule-based processing and emotion - as well as significant correlations between activations of the right amygdala and moral disgust ratings.

Following a social psychological approach, although people wish to protect their moral self-image (Monin & Jordan, 2009) and want to see themselves as moral actors (Mazar, Amir, & Ariely, 2008; Nisan, 1991), studies have also demonstrated that people recognize that moral concepts, like purity, can have different meanings depending on the type of social relationship a given situation involves (Rai & Fiske, 2011). According to what is known as *moral flexibility* (Bartels, 2008), contextual and societal factors could influence which values are more relevant than others. Morgan, Skitka, and Wisneski (2010) argued that different individuals may see some issues as relevant or irrelevant to morality. For example, people know that tax evasion is wrong, that it represents a risk because there is a certain probability of being discovered and punished, and that it leads to higher taxes for the remaining taxpayers. Empirical literature on the ethics of tax evasion reveals that, in general, there is strong moral opposition to it (e.g., Bosco & Mittone, 1997). However, the opposition to tax evasion could be weaker in a number of circumstances: cases in which the government engages in human rights abuses or cases in which the tax system is perceived as unfair (McGee, 2012). Which moral values become sacred for a group is somewhat arbitrary, a function of cultural context and need (Ginges, 2015). McGraw and Tetlock (2005) found that apparently absolute opposition to selling body organs on the marketplace was substantially reduced when, for example, it was emphasized that this trade would save many lives. In other words, what counts as a taboo trade-off varies dramatically across ideological subcultures, group-specific approaches to morality, and historical periods (Tetlock, Peterson, & Lerner, 1996). There are many opportunities for reframing issues that involve sacred values for groups. As in religions, values survive in time and in space because they are readily rewritable in ways that are sensitive to changing contexts (Atran & Norenzayan, 2004). The way in which moral values are relevant and sacralized could depend on collective reasons and could be based on a specific ingroup internalized conviction of right and wrong (Bartels et al., 2015). However, moral values can be defined as sacred by means of a taboo against material trade-offs, with several implications for intergroup dynamics and conflicts. What gives sacred values their meaning and their moral status is their separation from the profane domain of everyday life (Durkheim, 1955; Tetlock, 2003). Therefore, their defining characteristic is a taboo against valuing moral values along a material or monetary scale (Ginges, Atran, Medin, & Shikaki, 2007).

## Moral Sacredness and Political Ideology

The political arena is a field where moral convictions are the most debated and relevant for guiding the attitudes and behavior of electors, parties, and candidates. Discussions around inviolable values abound in many fundamental political disputes (e.g., The right to life, LGBT+ rights or the death penalty), and they often make disputes much harder to resolve. With regard to political issues, there is considerable variation in the degree to which people’s opinions are vested with moral conviction (e.g., Skitka, Bauman, & Sargis, 2005). Increased moral conviction about a given issue predicts increased prejudice toward those who have different views (Skitka et al., 2005), as well as distrust of political authorities who have to work on the issue (Wisneski, Lytle, & Skitka, 2009). Skitka and colleagues (2005) found that attitudes held with strong moral conviction predicted greater social and physical distance from, and intolerance towards, dissimilar others, lower levels of good will and engagement in attitudinally heterogeneous groups, and inability to generate procedural solutions to resolve disagreements.

Graham and Haidt (2010) argued that the most intractable political debates are likely to involve binding moral foundations, whereas the greatest degree of commonality may be found in issues related to individualizing ones. According to the Moral Foundations Theory (MFT; Haidt & Joseph, 2004; Haidt & Graham, 2007), individualizing foundations are related to care for and devotion to the discomfort of others (Harm / Care) and concern for justice and rights (Fairness / Reciprocity); while binding foundations describe the concern for and interest in social solidarity and responsibilities of group membership (Ingroup / Loyalty); social order, social role fulfillment and respect for traditions and institutions (Authority / Respect); and interest in control of impulses and desires (Purity / Sanctity). Individualizing foundations are described as the source of those moral intuitions correlated with the liberal philosophical tradition with its emphasis on the rights and welfare of individuals. Individualizing foundations are specific to a secular contractual society prioritizing individual rights and in which binding foundations are less important, or even elicit racism, prejudice or nationalism, authoritarianism, homophobia and disgust-based restrictions on the rights of women (Haidt & Kesebir, 2010). Binding foundations correlate with many conservative and religious moralities with their emphasis on group-binding loyalty (see also Graham & Haidt, 2012), and tend to be supported by more conservative elements within a society. In general, conservatives prefer a binding approach (i.e., They give similar evaluations on all five sets of moral foundations), whereas liberals show a preference for an individualizing approach (i.e., They give higher evaluations on fairness and harm moral foundations than on other foundations; Graham & Haidt, 2010; Graham, Haidt, & Nosek, 2009; Graham et al., 2011; Haidt & Graham, 2007; Haidt & Joseph, 2004). Testing the MF hypothesis in different countries, Graham and colleagues (2011) found that liberalism / left-wing ideology sustains liberty and equality as fundamental political goods, and so liberal people mainly support individual rights and the use of government programs to extend such rights as widely - and as equally - as possible. On the other hand, the fundamental values of conservatism / right-wing ideology are found in their propensity to preserve the status quo in human welfare and long-existing institutions, norms, and traditions across generations. Conservatives support solid institutions and social control as necessary and they tend to believe that they are hard to replace if delegitimized. However, these assumptions have not always been confirmed in all studies. For instance, Davis and colleagues (2016) could not generalize the moral foundations hypothesis which predicted that conservatism would positively correlate to binding foundations in black people in the United States.

Few studies used the Moral Foundation approach to investigate left-wing and right-wing moral reasoning in the Italian context (D’Alberti, 2013; Di Battista, Pivetti, & Berti, 2018; Milesi, 2017). Across three studies carried out in Italy from 2010 to 2013, Milesi (2017) found that the intention to vote for right-wing political groups rather than for left-wing rivals is associated with concerns about sanctity. Results also showed that individualizing is weakly related to political orientation, particularly in the dimension of care. Di Battista, Pivetti, and Berti (2018) found that both Italian left and right-wingers attribute higher scores to individualizing moral foundations than to binding ones. Right-wingers give similar evaluations on all five sets of moral foundations, whereas left-wingers give higher evaluations on individualizing foundations than on the other foundations. These studies did not measure moral foundations in a taboo trade-off framework.

In general, existing research measuring moral foundations in a taboo trade-off framework relies primarily on the Moral Foundations Sacredness Scale (MFSS; see Graham et al., 2009). The MFSS is designed to measure an individual’s willingness to violate moral norms in exchange for money (e.g., “Kick a dog in the head, hard”; Graham et al., 2009). As far as we know, only one Italian study administered the MFSS (D’Alberti, 2013) in order to study moral foundations and voting intentions in the Italian general election in 2013. The author found that the correlations between the indexes obtained with two methods of measurement (moral foundations questionnaire and MFSS) were quite different. According to the MFSS measurement, the only relevant result was an unexpectedly high foundation score for the political center. The Italian centrist faction seemed particularly sensitive to the notion of asking for money in exchange for violating taboos. D’Alberti (2013) highlighted that an explanation might lie in Italian political history. A centrist party such as Democrazia Cristiana (Christian Democracy) was traditionally the party with the closest ties to Catholics for a long period, namely, from the end of the Second World War until the 1990s. A strong identification with Catholic values for the centrist parties might explain these high scores in the moral trade-off measure, but D’Alberti cannot explain why these scores weren’t reflected in the moral foundations questionnaire.

Other studies, which aimed at measuring purity violations alone, have used scenarios involving incest and sex with a dead chicken (Feinberg, Willer, Antonenko, & John, 2012; Pennycook, Cheyne, Barr, Koehler, & Fugelsang, 2014), or vignettes involving incest and eating a pet dog (Eskine, Kacinik, & Prinz, 2011; Schnall, Haidt, Clore, & Jordan, 2008; Wheatley & Haidt, 2005). These specific scenarios and scales of moral compromise are difficult to adapt to Italian participants, as they are generally against these violations. For instance, in Italy incest and sexual relations between people and animals are punishable by law, therefore the moral applicability of these sentences, scenarios, and items is complicated. For this reason, we suspect that items designed to measure an individual’s willingness to violate moral norms in exchange for money as in previous studies will be particularly rejected by participants, obscuring any difference between groups.

## The Current Study

This study aims to better understand the moral quandaries people face when they debate sacred values and to further the study of moral psychology and its link with political ideology in the Italian context, showing 1) how compromise on moral foundations unveils the moral reasoning of the political left, center, and right in Italy; 2) which set of moral factors plays the most important role in these distinctions. The inclusion of the assessment of omission as a compromises on moral foundations is useful for understanding the moral roots of the ideological debates between groups. Furthermore, exploring moral psychology and judgments calls for different tools and methods that can cover all moral domains. For these reasons, we aim to use a new tool to measure the sacralization of moral foundations in a taboo trade-off context, drawing inspiration from the MFSS. We are interested in showing how a moral dilemma can conflict with the political groups’ internalized convictions of right and wrong in the Italian context. Moral regret for compromise on moral foundations in some specific dilemmas could explain what kind of morality a group and its members have. In line with the relevant research, MF hypothesis predict that conservatives prefer the binding approach (i.e., They give similar evaluations on all five sets of moral foundations), whereas liberals show a preference for individualizing moral foundations when compared to conservatives (Graham et al., 2009; Graham et al., 2011). Adapting Tetlock’s work on sacred values and taboo trade-offs (Tetlock et al., 2000) to make moral judgments “personal and visceral” (Graham et al., 2009, p. 1036) or personally relevant, Graham and colleagues (2009) showed participants’ resistance to contemplate a trade-off. Liberals refused to make trade-offs on individualizing foundations but were more willing to perform actions that violated the binding foundations. They were less likely than conservatives to see trade-offs related to binding foundations as violations of sacred values. The authors commented their results, finding that liberals mainly justify moral rules in terms of their consequences for individuals; they are familiarized with balancing opposing interests with the primary interest of individual care and rights. Conservatives, in contrast, are more likely not to break moral rules, even when the consequences would be positive, because they justify these values as handed down from authorities, such as God, or from earlier generations. The authors highlighted that this deontological reluctance to make trade-offs could be well detected by the methods used in the MFSS (Graham et al., 2009). The opportunity for moral compromise on sacred values, putting at risk those moral principles with which a group and its members strongly identify (Goodstein, 2000), could disclose which sets of foundations are truly inviolable for the left and the right. In this study and in line with the literature (Graham et al., 2009; Graham et al., 2011), it is hypothesized that Italian center - left morality relies heavily on the individualizing foundations that people would not compromise by accepting money in exchange for violating them. On the other hand, we predict that those on the center - right would be less likely to violate binding foundations for money than left-wingers. We also expect to find high scores on either individualizing or binding foundations for the political center, which can represent those Italian political parties traditionally linked to religiousness (D’Alberti, 2013).

Specifically, in the assessment of moral foundations via a new moral compromise foundation scale, namely the Omission as a Compromise on Moral Foundations scale (OC-MF), it is predicted that:

*H1*: Center and left-wing Italian participants would consider individualizing moral foundations as more relevant (i.e., Lower willing to compromise on them) than binding foundations.

*H2:* Center and right-wing Italian participants would consider binding moral foundations as more relevant (i.e., Lower willing to compromise on them) than left-wing Italian participants.

Confirmation of a two-factor structure of the OC-MF according to one of the principal two-factor models identified by Graham and colleagues (2011) is also expected.

## The Research Context

Italy has a multi-party system characterized by rapidly changing governments. It is recognized as a Westernized country with a modern liberal tradition with regard to the importance of the protection of each individual and his / her rights across every level of the political spectrum. Between 1996 and 2008, the Italian political parties were organized into two large center-left and center-right coalitions. In 2013 the bipolar system was unsettled by several new political actors and widespread political disaffection increased among Italian citizens (Pasquino & Valbruzzi, 2013). However, despite the alleged end of ideologies (Bell, 1965) and despite the disaffection for specific parties, in Italy the relevance of the left-liberal and right-conservative distinction is still present in the electors’ identification and represents a valuable organizing principle of the political space (Baldassarri, 2013; Bellucci & Maraffi, 2014; Corbetta, Cavazza, & Roccato, 2009; Gries, 2017; Natale, 2006; Vegetti, Poletti, & Segatti, 2013). Furthermore, due to growing political party fragmentation and unstable coalitions between parties, personal reasons (such as moral values or the vision of a “good society”) increase in importance in people’s personal political orientation and identification, more significantly than previous traditional indicators for political decision-making (such as geographical derivation, status, education, and occupation; Caprara, Schwartz, Capanna, Vecchione, & Barbaranelli, 2006; Lakoff, 2004; Vecchione, Tullio, & Caprara, 2011; Westen, 2007).

Italian political leaders often invoke sacred values in their electoral programs and use them to discredit political adversaries. The sacralization of the same moral imperative (i.e., The right to life) makes discussion between political groups particularly unfeasible. Indeed, moral issues are often the focus of severe electoral debates on the inviolability of certain issues.

## Method

### Participants

The convenience sample included 224 Italian participants recruited via snowball sampling with the collaboration of research assistants. The majority (58.6%) were females (males 37.6%; 4% gender information missing). Their mean age was 27.93 years (*SD* = 10.9; range 18-75). About 59.8% of the participants were students. As for political orientation (*M* = 4.1; *SD* = 1.5), the sample was fairly balanced between left, right, and center, revealing that 27.7% of the participants positioned themselves on the left of the left-right axis (from 1 to 3 on the scale), whereas 27.7% positioned themselves in the center (point 4 on the scale), and 30.4% positioned themselves on the right (from 5 to 7) (14.3% did not report their political orientation). Participants who declared their political party affiliation were a minor part of the total sample (*n* = 82; 36.6%).

### Procedure

The research was carried out by the University G. d’Annunzio of Chieti-Pescara in Italy. The questionnaire was implemented using a Google Drive Form and administered in the Italian regions of Abruzzo and Lazio. Participants were recruited via snowball sampling with the collaboration of researchers and doctoral students. They posted the questionnaire link on social networks or sent it to the mailing lists of both close (e.g., Friends) and more distant acquaintances (e.g., Colleagues) who were involved in political groups or who they deemed to be very interested in political debates. The questionnaire took approximately 20 minutes to fill in.

The research complied with the Code of Ethics of the Italian Psychology Association (Associazione Italiana di Psicologia [A.I.P.], 2015).

### Measures

#### The Omission as a Compromise on Moral Foundations Scale (OC-MF)

Individuals’ willingness to omit/neglect moral norms in exchange for money was measured. Following on from studies relating to moral taboo trade-offs (Graham et al., 2009; Graham & Haidt, 2012; Tetlock et al., 2000) and inspired by the Moral Foundations Questionnaire (MFQ; Graham et al., 2009; Graham et al., 2011), we generated items to measure compromise on moral foundations. Each item measures the violation of moral foundations in exchange for money. In the MFSS by Graham and colleagues (2009), five potential taboo violations for each moral foundation were administered. For example, the authors asked participants how much money someone would have to pay them to: “Kick a dog in the head, hard” (for Harm); “Renounce their citizenship” (for Ingroup); “Get a blood transfusion from a child molester” (for Purity). In order to test the applicability of the MFSS in the Italian sample (Graham et al., 2009), we ran a pilot study adapting D’Alberti’s (2013) Italian translation of the MFSS. The scale was administered to a sample of 23 Italian undergraduate psychology students who filled in the questionnaire during classwork. They were invited to carefully read, fill in and critically evaluate the scale on their own. Then, students were invited to discuss their personal evaluation in groups of four/five students each. After that, they were asked to answer to a semi-structured interview concerning the main strong and weak points of the scale. The results suggested that the scale might be inadequate to measure sacred values in the Italian context, both in individuals and in groups. In particular, three problems emerged from the students’ answers: 1) social desirability: many items of the scale refer to unacceptable issues for Italian culture (e.g., “Stick a pin into the palm of a child you don’t know”); 2) MFSS items measure some aspects that in Italy are considered crimes, antisocial behavior or cruelty rather than moral concerns (e.g., “Kick a dog in the head, hard”); 3) some items are not suitable for the morality of Italians, as they are not as nationalistic as citizens of other countries (e.g., “Curse the founders or early heroes of your country (in private, nobody can hear you)”). Similarly, Davis and colleagues (2016) found that the specific items on the moral foundations questionnaire assessing loyalty to one’s country and loyalty to one’s family may not be strongly aligned in some subsamples. For example, among black people in the United States, some individuals may feel very loyal to their family and community, but feel less loyalty towards their country. This might be true in Italy as well.

However, the moral trade-off framework appeared to us very suitable for measuring the moral inviolability that makes moral foundations sacred. Graham and colleagues (2009) argued that the MFSS is a measure that makes moral judgments more “personal and visceral” (p. 1036) than in other instruments used in MF theory and studies, eliciting personally relevant responses by presenting subjects with moral trade-offs. We generated potential compromises on moral foundations in a moral trade-off framework, partially drawing inspiration for the wording from the moral relevance scale of the MFQ (reported in Graham et al., 2009, Appendix A). This MFQ scale offers a decontextualized method that is appropriate for assessing moral values as values that are said to be generalized across contexts (e.g., “When you decide whether something is right or wrong, to what extent are the following considerations relevant to your thinking: Whether or not someone acted unfairly [for fairness]; Whether or not someone violated standards of purity and decency [for purity]”; Graham et al., 2009). Drawing inspiration from this scale, 17 items to measure compromise on moral foundations were generated (6 items for individualizing - 2 items for fairness and 4 for harm, and 11 items for binding - 3 for ingroup, 3 for authority, 5 for purity). Participants first read: “How much money would someone have to pay you not to…”. They then rated items (e.g., “To show compassion towards those who suffer” [for Individualizing]; “To be clean and fresh” [for Binding, see Table 1]) using a specific response scale that measures an omission rather than active behavior. Participants were asked to evaluate items on a 7-point scale ranging from 1 = *I would not agree to do it for money,* to 7 *= I would agree to do it even for less than 10 Euros* (see Table 1). We expected people to be more willing to accept a financial offer for a transgression when the sacred values are presented as passive as opposed to active behavior (Ayanian & Berwick, 1991; Baron & Ritov, 2004; Feinberg, 1984). Because of the “omission bias”, transgressions caused by omission or inaction have been found to be less relevant than those that are caused by action (Ayanian & Berwick, 1991; Baron & Ritov, 2004; Feinberg, 1984). Similarly, according to the “action principle” (Cushman, Young, & Hauser, 2006), it is easier for people to abstain from moral behavior than to explicitly refuse to act morally. Active and passive transgressions are treated differently by the law (Feinberg, 1984) and are judged and weighed differently by spectators (e.g., Baron & Ritov, 2004; Spranca, Minsk, & Baron, 1991), producing different effects of social desirability. For instance, participants are more likely to serve their self-interest by refraining from telling the truth (lie of omission) than by deliberately lying (lie of commission) when they face the temptation to benefit from dishonesty (Pittarello, Motro, Rubaltelli, & Pluchino, 2016).

#### The Moral Relevance Scale

Twenty-three assessments of the moral relevance scale of the MFQ collecting participants’ evaluation of the abstract concepts of moral foundations (see Graham et al., 2009) were administered as well. Participants first read: “When you decide whether something is right or wrong, to what extent are the following considerations relevant to your thinking?”. They then rated moral relevance items using a 7-point scale (from 1 = *not at all*; to 7 = *very much*).

#### Political Ideology

Participants’ political orientation was assessed on a 7-point scale ranging from 1 (= *Left*) to 7 (= *Right*). A simple one-dimensional spectrum of left–right or liberal–conservative represented political orientation well (Corbetta et al., 2009; Jost, 2006; Jost, Glaser, Kruglanski, & Sulloway, 2003). Haidt and Graham (2007) confirmed the fact that political ideology and orientation can be self-assessed and that the unidimensional left-right construct has common meaning across cultures, despite differences in political and historical background.

One open-ended question assessed participants’ affiliation with a specific political party within the Italian political context (“Which political party do you vote for?”).

The socio-demographic section probed participants’ age and gender.

## Results

### Confirmatory Factor Analyses

To evaluate the adequacy of the OC-MF, we performed a Confirmatory Factor Analysis (CFA) on all 17 items, and re-specified the model by looking at the standardized residual covariances between the items (Jöreskog & Sörbom, 1984): large values (above 2 in absolute value) were used for indicating that the model was not correct. Therefore, the items with largest standardized residual covariances were discarded one by one while looking at the improvement in goodness-of-fit indices. The final model included 14 items: three items for each dimension of care, ingroup, authority and purity, and two items for fairness (see items in Table 1).

**Table 1:** Corrected Item-Total Correlations

OC-MF subscale	Individualizing	Binding
Harm		
H1: To show compassion towards those who suffer	.52	
H2: To care for those who suffer	.62	
H3: To listen to someone who suffers	.63	
Fairness		
F1: To be correct and honest	.65	
F2: To be fair	.67	
Ingroup		
I1: To be loyal to your family		.39
I2: To be loyal to your friends		.46
I3: To be loyal to your country		.60
Authority		
A1: To respect your cultural traditions		.68
A2: To respect social hierarchies		.63
A3: To obey your authorities		.72
Purity		
P1: To be faithful		.42
P2: To be clean and fresh		.40
P3: To be pure in your soul		.49

*Note.* OC-MF = Omission as a Compromise on Moral Foundations Scale.

**Figure 1:**
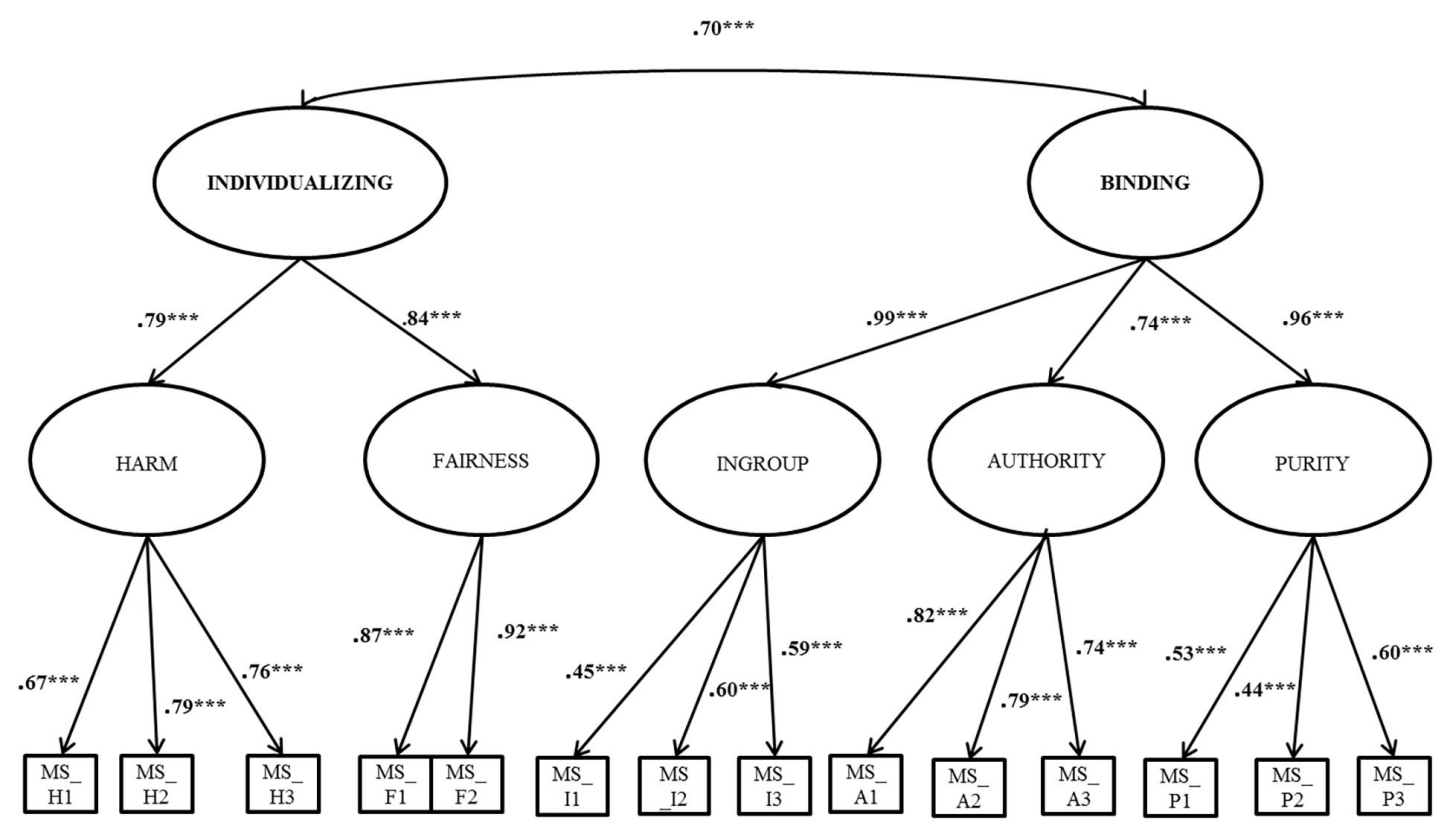
Hierarchical model for the OC-MF. ***p < .001.

The model fit of the hierarchical models containing two superordinate factors (individualizing and binding) was assessed using IBM SPSS AMOS 25.0 software. The fit indices showed an acceptable model fit (χ^2^ = 174.16; *df* = 72; *p* < .001; χ^2^/*df* = 2.42; CFI = .91; RMSEA = .08; Bentler, 1995). The correlation between the two superordinate factors was significant and positive (*r* = .70; *p* < .001), and the factor loadings were highly significant (*p* < .001) (see Figure 1). To test an alternative model with a single superordinate factor, a second CFA was run. Fit indices on an alternative CFA model with a single superordinate factor were not as good (χ^2^ = 196.54; *df* = 73, *p* < .001; χ^2^/*df* = 2.69; CFI = .89; RMSEA = .09) and therefore, the first model with two superordinate factors was retained.

With regard to individualizing, the *Corrected Item-Total Correlations* ranged from .52 to .67; with regard to binding, the *Corrected Item-Total Correlations* ranged from .39 to .72 (see Table 1). These coefficients showed that all of the individual items were associated with their respective subscale and were higher than the rule of thumb minimum value of 0.2 (Kline, 1986). Internal consistency was examined using *Cronbach’s Alpha* computed for the two subscales. *Means, Standard Deviations*, *Cronbach’s Alpha*, and *Inter-Correlations* between individualizing and binding foundations measured with the OC-MF and the moral relevance scale are reported in Table 2.

**Table 2:** Means, Standard Deviations, Reliabilities, and Inter-Correlations Between the Measures

Measure	*M*	*SD*	α	1	2	3	4
OC-MF							
1. Individualizing	6.25	0.98	.82	1	.53	.39	.28
2. Binding	5.84	1.08	.82		1	.27	.54
Moral Relevance							
3. Individualizing	6.10	0.80	.90			1	.47
4. Binding	5.28	1.09	.85				1

*Note*. All correlations are significant, *p* < .001.

All the items of the scale were reversed. Firstly, the items for the 5 subscales of harm, fairness, ingroup, authority and purity were averaged; secondly the average of the subscales harm and fairness built the individual foundation scale, and the average of the ingroup, authority and purity subscales built the binding foundation scale.

The individualizing and binding foundations, assessed through the OC-MF, were related to the same constructs measured with the moral relevance instrument (individualizing: *r* = .39; *p* < .001; binding: *r* = .54; *p* < .001), even if the correlation for individualizing was only of medium effect size, and thus of low convergent validity. Individualizing and binding measures were related both in the OC-MF (*r* = .53; *p* < .001) and in the moral relevance scale (*r* = .47*; p* < .001), and the difference between these two correlations was not significant (*Cohen’s q =* .08; see Cohen, 1988). Political orientation was related to binding foundations, both in the OC-MF (binding: *r* = .25; *p* < .001) and in the moral relevance scale (binding: *r* = .27; *p* = < .001), and the difference between these two correlations was not significant (*Cohen’s q =* .021); individualizing foundations were not related to political orientation, either in the OC-MF (individualizing: *r* = -.03), or in the moral relevance scale (individualizing: *r* = -.04).

### Hypotheses Testing

**Figure 2:**
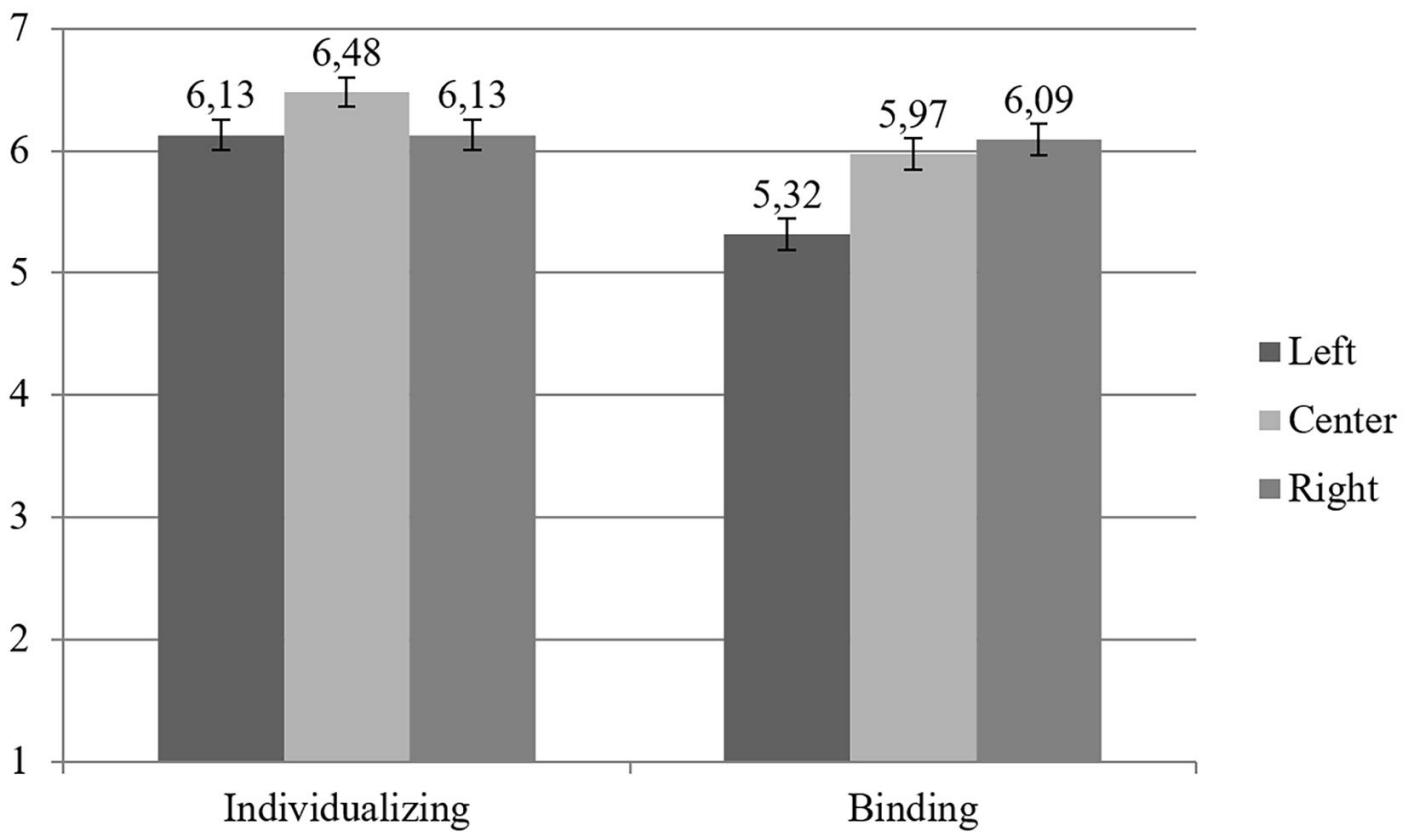
Individualizing and binding moral compromise foundations according to the level of political orientation. *Note.* Higher scores indicate greater aversion to compromise on moral foundations. Error bars represent 95% confidence intervals.

In order to test hypotheses 1 and 2, a Repeated Measures Analysis of Variance (ANOVA), with political orientation (left, center, and right) as a between-subjects factor and moral compromise foundations (individualizing and binding) as a within-subject factor was run. The ANOVA yielded a significant main effect for moral compromise foundations, *F*(1, 189) = 40.89, *p* < .001, $\eta_{p}^{2}$ = .18, showing that all the participants refused to compromise more on individualizing (*M* = 6.25; *SD* = 0.97) than on binding moral foundations (*M* = 5.79; *SD* = 1.09), and a significant main effect of political orientation, *F*(2, 189) = 5.71, *p* = .004, $\eta_{p}^{2}$ = .06, indicating that center and right-wingers endorsed a greater aversion to compromise on all moral foundations (*M*_center_ = 6.23; *SD* = 0.91; *M*_right_ = 6.11; *SD* = 0.92) than Left-wingers (*M*_left_ = 5.73; *SD* = 0.78). Moreover, these main effects were qualified by a reliable interaction between moral compromise foundations and political orientation, *F*(2, 189) = 10.12, *p* < .001, $\eta_{p}^{2}$ = .10. A simple effect analysis showed that center and left-wingers refused to compromise more on the individualizing than on binding moral foundations, Left: *F*(1, 189) = 42, *p* < .001; $\eta_{p}^{2}$ = .18; Center: *F*(1, 189) = 16.78, *p* < .001; $\eta_{p}^{2}$ =.08, whereas a non-significant effect emerged with regard to right-wingers, *F*(1, 189) = 0.12, *p* = .73; $\eta_{p}^{2}$ = .001, supporting *H1* (see Figure 2). Furthermore, in line with *H2,* a significant effect for moral compromise binding foundations emerged across the levels of political orientation, *F*(2, 189) = 10.08, *p* < .001; $\eta_{p}^{2}$ =.10, whereas the effect for moral compromise individualizing foundations across the levels of political orientation was non-significant, *F*(2, 189) = 2.86, *p* = .06; $\eta_{p}^{2}$ = .03. Left-wing participants indicated lower aversion to compromise on binding moral foundations than any other study group (Center: *p* = .001; Right: *p* < .001), whereas center and right wingers showed no difference (*p* = .51), supporting *H2* (see Figure 2).

## Discussion

The study explored some types of compromise on sacred or inviolable moral foundations across different political orientations in Italy. The OC-MF, a new measure based on omission as a part of compromise on moral foundations, was introduced. The Moral Foundations perspective (Graham et al., 2009; Haidt & Graham, 2007; Haidt & Joseph, 2004) guided the development of the instrument, which was designed not only to show the relevance attributed to a particular set of moral foundations in relation to political orientation in Italy, but also to identify the “sacralization” of individualizing and binding foundations using an innovative taboo trade-off framework. Its factor structure confirmed the presence of two dimensions of moral compromise foundations, namely individualizing and binding. The OC-MF also fairly corresponded to the existing scale of moral relevance found in the MFQ. The instrument we put forward has proved to be consistent with the moral foundations hypothesis. The findings showed that Italian center- and left-wing participants consider individualizing moral foundations as more relevant and inviolable than binding foundations. Italian center and right-wing participants consider binding moral foundations as more relevant and inviolable than Italian left-wing participants. Italian participants positioned at the center of the political spectrum were found to show a binding approach that was similar to right-wingers, but also high levels of individualizing foundations, comparable to those of left-wingers, in line with the previous Italian study by D’Alberti (2013). A strong identification with Catholic values for center parties might explain the relevance attributed to binding foundations, because these foundations in fact tend to be reinforced by the most conservative and religious elements within a society (see also Graham & Haidt, 2012). According to Bobbio, Matteucci, and Pasquino (1976), those participants from the center could represent either the moderate area of politics or the undecided voters. In Italy, the area of the political center is characterized by dissimilar political affiliations and parties ranging from left to right.

Furthermore, it was found that everyone’s morality relies heavily on individualizing foundations. In other words, the findings suggested that left, center, and conservatives express particular aversion to compromise on justice issues and care for the discomfort of others. The results challenged previous findings which showed that liberals care more than conservatives about individualizing issues (Graham et al., 2009, Studies 1 and 2), but were in line with studies which measured “sacredness” reactions to taboo trade-offs (Graham et al., 2009, Study 3). In line with Graham and Haidt (2012), concerns about the rights of individuals and their freedom seem so widespread that they might be said to be universal. Also in the Italian context, individualizing moral foundations could represent a shared point of view between left- and right-wingers. Milesi (2017) argued that in systems which are not perfectly bipolar, and in fragmented and unstable electoral contexts, such as Italy, voters who have seemingly opposite political positions do share some moral concerns.

In general, the OC-MF offers new understanding of moral reasoning with regard to the transgression of sacred values in terms of passive behaviors. Choosing to actively act in order to violate a moral norm can elicit a strong negative social and emotional response, inhibiting considerations of a compromise on moral foundations. On the contrary, we assumed it to be easier for people to contemplate the mere opportunity of a compromise on moral foundations when they can choose to omit moral behaviors (Baron & Ritov, 2004). This is in line with Cushman and colleagues’ (2006) finding that participants viewed harm-inflicting acts of omission as more permissible than harm-inflicting acts of commission. Furthermore, the OC-MF framework has the potential to bring out the inviolability of some moral values on which it is impossible to compromise, making debates so difficult between different political orientations in Italy.

### Implications

Disagreements over sacred values, particularly those associated with political ideology, abound in many public debates, making disputes very hard to resolve (Bazerman, Tenbrunsel, & Wade-Benzoni, 2008; Susskind et al., 2005). In line with Argo and Ginges (2015), regardless of whether sacred values are openly elicited in a group discussion, they must be acknowledged and considered in political debates. Good strategies for negotiations involve understanding how sacred values can affect decision making. Understanding moral sacredness associated with a given issue is relevant in order to suggest possible spaces and limits regarding negotiations (Atran & Axelrod, 2008). Liberals and conservatives are divided on many issues, including abortion, LGBT+ rights, women’s rights, gun ownership and more, and such differences help to explain many of the contrasting stances the two sides take (Brandt, Reyna, Chambers, Crawford, & Wetherell, 2014; Caprara et al., 2006; Graham et al., 2009). However, our findings suggested that in a taboo trade-off framework, individualizing moral foundations could be equally inviolable for left, center, and right, and in this sense this common point of view could serve as counterweight in political debates. This is partially in line with Schein and Gray’s (2015) finding that, despite moral diversity regarding specific issues (e.g., Patriotism, chastity), moral cognition in both liberals and conservatives is rooted in a harm-based template. Moreover, our findings also suggested the consideration that a compromise on binding moral foundations is seen differently by left, center, and right-wing participants, and thus sacred positions relating binding foundations may be representative of the most intractable political debates (in line with Graham & Haidt, 2010).

Gibson (2011) found that one of the greatest potential problems causing impasse in decisions involving sacred values is that parties do not consider the extent to which some values are “priceless” for the competing sides, and said sides are not willing to work within those constraints (see also Argo & Ginges, 2015). However, the literature also suggested that there are ways that people across the ideological spectrum can make their stance understandable to a person from the other side. For instance, Voelkel and Feinberg (2018) found that a moral reframing technique can be used to overcome the rigid stances partisans often hold and help to develop political acceptance. Furthermore, a capacity for contextual understanding of moral concerns is important in order to solve complex social problems when multiple moral reasonings are conflicting, such as in the case of abortion or LGBT+ rights. This capacity is also important for living in groups with different moral values or in groups that prioritize the same moral values differently (Atran & Axelrod, 2008). Skitka, Washburn, and Carsel (2015) found that people do not accept or expect their moral convictions to be contextually contingent or situationally variable, and tend to be offended by the idea that morality can be relative. For example, people morally convinced that abortion is wrong are likely to believe it is objectively and universally wrong and that it should be universally banned. In the context of international political negotiations, Argo and Ginges (2015) argued that this rhetoric reinforces absolutism, turning decision making into an irresolvable conflict in which people inflexibly refuse to manage creative solutions. Similarly, in the political field, Zagrebelsky (2008) described two different or even conflicting moral approaches. The first is a strict approach that leads to a rigid defence of moral values, unlikely to be negotiated or reframed depending on the context. For instance, the “right to life” could be sacralized and abortion would therefore be forbidden in all cases. On the basis of the second perspective, moral issues are rewritable in accordance with each specific and concrete situation. In this case, abortion could be viewed as a restriction of the right to life of the fetus that is opposed to and conflicting with the woman’s right to health care; according to an attentive evaluation of these two conflicting situations, the second perspective generally prevails and States are obliged to respect, protect, and fulfill the woman’s right, as, unlike a fetus, she is already a person.

Drawing inspiration from Gibson (2011), Argo and Ginges (2015) suggested that for a successful political negotiation it is necessary to be aware of when individuals are conflating a sacred position with sacred values. A conceptual clarification could allow for the reframing of sacred values so that they are preserved even in the face of a moral compromise. The authors argued that a good negotiation process emphasizes that sometimes a compromise is the only way one could retain at least some portion of the sacred. A complete discussion of those strategies enabling individuals and groups to negotiate on sacred values is beyond the scope of this paper, however some examples concerning the Italian specificities may provide constructive suggestions. Over the course of more than thirty years, one of the most violent political quarrels on issues with moral implications was focused on LGBT+ rights. In May 2016, the Italian Parliament gave its final approval to a law providing same-sex couples with many of the rights arising out of marriage (Gazzetta Ufficiale della Repubblica Italiana, 2016). The proposal supported by the Prime Minister Matteo Renzi and the left-wing coalition was postponed several times as a result of obstructionist opposition on the part of conservative and Catholics forces, who presented a barrage of amendments against the law (Tonacci, 2014). Italian conservative parties accused left-wing parties of not protecting the interests of the “natural family”, sacred values that are implied in the binding approach. Luigi Manconi, who was the President of the Italian Human Rights Commission, argued that the impasse in the approval of a law was mostly due to a debate between all the opposite parties that do not get in the right common perspective: the equal dignity of persons (Tonacci, 2014). A compromise was possible after a long negotiation on the need to distinguish same-sex partnerships, social units compliant with Articles 2 and 3 of the Italian Constitution, from families as natural gatherings as per Article 29 of said Constitution. This reframing enabled a compromise because both parties could adopt a version of their position which left their sacred values unchanged. For instance, the left-wing coalition emphasized the importance of the new law, stating that it was better than the previous arrangement and reprioritizing the issue of a full regulation of LGBT+ issues for the near future. The right-wing coalition and Catholic forces changed their sacred position against same-sex unions, accepting a norm where a compromise over their sacred values of the traditional family would no longer be necessary. A breakthrough was reached by recognizing that the political positions were not merely conflicting, but ultimately complementary when viewed in the broader perspective of long-term societal success.

### Limitations

The study had some important limitations that must be kept in mind when interpreting the results. One limit is the correlational nature of the research and the low representativeness of the convenience sample, mainly made up of participants from central and southern Italy. Future research should explore compromise on moral foundations in a more extensive Italian sample. We failed to insert a new measure of political affiliation. The majority of participants refused to report the party they do or would vote for. In view of the rapid evolution of the Italian political context, the widespread political dissatisfaction, and the lack of confidence in the party system that was observed in other studies (Bellucci & Maraffi, 2014; Martini & Quaranta, 2015), we should add new measures of political ideology and political disaffection. Furthermore, in future studies a sixth moral foundation should be explored, namely Liberty / oppression (Iyer, Koleva, Graham, Ditto, & Haidt, 2012), describing the feelings of resentment people feel toward those who dominate them and restrict their liberty.

### Conclusion

In this study, moral foundations were measured in a taboo trade-off framework among Italian participants, finding that political orientation correlates with moral foundations as sacred values. The new scale shows the hypothesized differences between Italian political groups: the left prioritizes individualizing foundations as opposed to binding ones in a context of moral dilemma, whereas conservatives and centrists have a broader idea of sacredness. This deep understanding of the opponents’ sacred values could offer new opportunities to improve ideological debates in the political arena. Although there is still much to learn about what leads people to have strong sacred values and about the full range of consequences of these attitudes, these results point towards a deeper understanding of the role that moral concerns may play in different ideological viewpoints in Italy.
